# Phase angle and triglyceride-glucose index as biomarkers of glycemic control, body composition and cardiovascular risk in adolescents with type 1 diabetes: a cross-sectional study

**DOI:** 10.1007/s40618-026-02969-2

**Published:** 2026-06-29

**Authors:** Marta García-Poblet, José Miguel Martínez-Sanz, Manuel Antonio Alberola-Chazarra, Esther Soler-Climent, Ana Pilar Nso-Roca, Isabel Sospedra

**Affiliations:** 1 Nursing Department, Faculty of Health Science, University of Alicante, 03692 Alicante, Spain; 2 Research group on Applied Dietetics, Nutrition and Body Composition (DANuC), University of Alicante, 03692 Alicante, Spain; 3 Pediatrics Unit, General Hospital of Elche, 03203 Elche, Alicante, Spain; 4 Research and Innovation Area, Health Department of General Hospital of Elche, Elche, Alicante, Spain; 5 Pediatrics Unit, University Hospital of San Juan, 03550 San Juan de Alicante, Alicante, Spain

**Keywords:** Adolescent, Biomarker, Metabolic risk, Phase angle, Triglyceride-glucose index, Type 1 diabetes mellitus

## Abstract

**Purpose:**

Traditional markers such as HbA1c and BMI are commonly used to assess metabolic risk in adolescents with type 1 diabetes mellitus (T1DM), but novel biomarkers like the triglyceride-glucose (TyG) index and phase angle (PA) may provide a more comprehensive evaluation of metabolic risk. However, their potential role in this population remains unclear. We aimed (1) to evaluate the association of the TyG index and PA with glycemic control, cardiovascular risk and body composition in adolescents with T1DM, and (2) to determine optimal cut-off points for these biomarkers to predict metabolic risk.

**Methods:**

Seventy-three adolescents (46.6% female, 10–19 years) with T1DM from two hospitals were included in this cross-sectional study. Sociodemographic, bioelectrical impedance (BIA) and clinical data were collected during routine visits. The TyG index was calculated from fasting triglyceride and glucose levels, and PA was measured via BIA. Correlations were analyzed using Pearson or Spearman coefficients, and ROC curve analyses were used to determine optimal cut-off points.

**Results:**

PA correlated positively with skeletal muscle and fat-free mass and inversely with cardiovascular risk indicators. TyG was strongly associated with adiposity, glycemic variability and cardiovascular risk markers. A preliminary TyG threshold of 8.09 was identified, while no PA cut-off could be established.

**Conclusions:**

PA and the TyG index are promising, non-invasive biomarkers that complement traditional measures in assessing metabolic risk in adolescents with T1DM. Integrating these biomarkers into routine evaluation may enhance early risk detection and support holistic management in this population.

**Trial registration number:**

NCT07021326.

## Introduction

Type 1 diabetes mellitus (T1DM) is a chronic autoimmune disorder characterized by the pancreatic β-cells destruction, leading to insulin deficiency and hyperglycemia [1]. Globally, over 1.2 million children and adolescents are affected, with incidence rising 3% annually [1]. According to International Society for Paediatric and Adolescence Diabetes (ISPAD), prevalence is projected to reach 13.5–17.4 million by 2024 [1].

The World Health Organization (WHO) defines adolescence as the period between 10 and 19 years of age [2] and this developmental stage is marked by significant physiological and psychosocial changes [2], making T1DM management particularly challenging due to variable treatment adherence and its impact on metabolic control and body composition [3].

Simultaneously with the growing prevalence of T1DM, obesity has also increased among these patients [4]. This may relate to insulin therapy, reduced physical activity, and behavioural factors such as emotional eating [4]. The relationship between obesity and T1DM appears to be reciprocal: weight increase is an adverse effect of T1DM therapy and, at the same time, constitutes a risk factor for its development [4]. In both cases, these conditions seem to be interconnected, which is significant when evaluating the role of body composition [5] and growth indicators, such as percentile and z-score, in routine assessments of adolescents based on their age and sex [5, 6].

In routine clinical practice, monitoring of T1DM relies on dietary assessment, body mass index (BMI), and glycated haemoglobin (HbA1c) [7]. HbA1c, the gold standard for long-term glycemic control [8], is strongly associated with vascular complications [8].

Current ISPAD guidelines recommend HbA1c to be < 6.5% in adolescents using advanced technologies such as continuous glucose monitoring (CGM), or ≤ 7.0% in cases where access to these technologies remains limited [8]. Although, HbA1c does not capture glycemic variability or hypoglycemic episodes [9, 10], CGM systems address this limitation by providing metrics such as the coefficient of variation (CV) and time in range (TIR), enabling a comprehensive evaluation of glucose variability and stability [8, 10]. The American Diabetes Association (ADA) advocates for the utilization of CV as the standard deviation normalized by the 24-hour average glucose concentration [11, 12], with a recommended target of ≤ 36% [8], and delineates TIR as the proportion of time spent within the target glucose range (70–180 mg/dL) over a 24-hour timeframe [12], with an optimal target of ≥ 70% [8].

Beyond traditional markers, novel biomarkers like the triglyceride-glucose (TyG) index and phase angle (PA) may provide a more comprehensive view of metabolic risk and are of particular interest [13, 14]. The TyG index, derived from fasting triglyceride and glucose [15], has been associated with insulin resistance, cardiovascular rick and glycemic control in type 2 diabetes [16, 17], adults with T1DM [18] and metabolic syndrome [19, 20]. However, its broader utility in adolescents with T1DM remains underexplored, warranting further investigation.

PA, obtained from bioelectrical impedance analysis (BIA) [21], is a non-invasive measure of cellular membrane integrity and fluid distribution [7], thereby representing variations in both the quantity and quality of soft tissue mass [21] and being considered one of the most reliable indicators of cellular membrane function [21]. Lower PA has been linked to poor prognosis, increased mortality and compromised nutritional status [22]. Although investigated in cardiovascular disease and other conditions [21, 23], its relevance in adolescents with T1DM still insufficiently studied, limiting its clinical and epidemiological applicability in this population.

Given the interplay between T1DM, obesity and cardiovascular disease, integrating TyG index and PA into clinical practice may provide a more comprehensive understanding of metabolic risk in adolescents with T1DM. However, specific cut-off values for these markers in this population have not yet been established. In this context, we aim (1) to evaluate the association of the TyG index and PA with glycemic control, cardiovascular risk, and body composition in a sample of adolescents with T1DM and (2) to determine optimal cut-off points for the TyG index and PA that predict metabolic risk in this population. We hypothesize that both the TyG index and PA are significantly associated with these parameters and may serve as useful biomarkers for metabolic risk assessment.

## Materials and methods

### Design

This is a cross-sectional study conducted in the paediatric units of the General Hospital of Elche and the San Juan Hospital (Alicante, Spain). The sample size calculation was performed with RStudio software (version 3.15.0, RStudio Inc., Boston, MA, USA). The significance level was set a priori at *p* = 0.05. The standard deviation (SD) for HbA1c percentage was determined from previous studies (SD = 1.26) [24]. Based on this methodology, the minimum required sample size was 65 participants, with an estimated error (d) of 0.30 for HbA1c percentage within a 95% confidence interval (CI). The study aimed for a statistical power greater than 0.80, achieving a calculated power of 0.96, which is considered high. In addition, the study design as well as the development of the manuscript followed the STROBE-nut guidelines for nutritional epidemiology, an extension of general STROPE for observational research.

### Study population

In both cases, data were collected from a sample of adolescents and young adults with T1DM after their routine consultations. The inclusion criteria required participants to be patients with T1DM aged between 10 and 20 years, with or without a continuous glucose monitor. The exclusion criteria specified that participants should not be hospitalized or pregnant, in the case of female patients.

### Data collection

Data collection was performed in person by two trained healthcare professionals from July 2022 to October 2024 during the patients scheduled routine consultations. Sociodemographic and clinical variables were collected directly from the patient’s clinical records. The biochemical parameters of interest were collected from most recent blood tests.

### Variables

#### Outcome variables: PA and TyG index

The PA was obtained using BIA with the portable device Biody-Xpert (Aminogram SAS, La Ciotat, France). The TyG index was calculated as Ln[fasting triglycerides (mg/dL) x fasting glucose (mg/dL)/2] [15].

#### Predictive variables

##### Sociodemographic and anthropometric variables

Body mass and stretch stature were measured during the consultation using a calibrated scale and stadiometer (SECA 704 s, Hamburgo, Germany). BMI, percentile and z-score (both based on BMI for age) were calculated using the WHO AntroPlus software 1.0.4 from the World Health Organization. Body mass was categorized as follows: underweight if percentile was less than 5, a normal weight percentile of between 5 and less than 85, an overweight percentile of between 85 and less than 95 and an obesity percentile of 95 or more [25]. Similarly, an underweight z-score of less than − 2, a normal weight z-score of between − 2 and 0.99, an overweight z-score of between 1 and 1.99 and an obesity z-score of more than 2 were assumed [26].

##### Bioimpedance variables

BIA data were collected using a standardized protocol [27]. Following the manufacturer’s instructions, participants held the device with the right hand and placed it on the right heel after cleaning contact areas with alcohol; measurements were obtained 10 s after activation. Variables recorded included PA (°), fat mass (Kg and %), fat-free mass (Kg and %), lean mass (Kg and %), skeletal muscle mass (Kg, and %) and several indices (calculated relative to stretch stature squared) and ratios: fat mass index, fat-free mass index, lean mass index, skeletal muscle mass index, fat mass/lean mass ratio and adipose-muscular index [28, 29]. Higher fat-free mass, lean mass and skeletal muscle mass indexes are considered beneficial, while lower fat mass index and adiposity-related ratios indicate healthier body composition [28, 29]. Data on total body water (TBW), extracellular water (ECW), intracellular water (ICW) and their ratios (ECW/TBW and ECW/ICW) were also collected.

##### Glycemic control variables

CV and TIR data were extracted from the records provided by the continuous glucose monitor, representing the mean values over the last 3 months in patients who had continuous glucose monitoring. Regarding HbA1c, it was measured during the medical appointment using the Alere Afinion AS100 Analyzer (Abbott, Illinois, United States) device.

##### Biochemical variables

Biochemical variables obtained by routine blood samples included: Glucose (mg/dL), HbA1c (%), total cholesterol (mg/dL), HDL cholesterol (mg/dL), LDL cholesterol (mg/dL) and triglycerides (mg/dL). From these parameters some ratios and indexes were calculated, including total cholesterol/HDL ratio, the LDL/HDL ratio, the triglycerides/HDL ratio [30–32] and the TyG index [15].

### Ethics

The study was conducted in accordance with the Declaration of Helsinki. Ethical approvals were obtained from the Research Ethics Committee of General Hospital of Elche (registration code PI 58/2024) and Research Ethics Committee of San Juan Hospital (committee no. 22/038). Both participants and their legal guardians were informed of the objective of the study and legal guardians signed an informed consent form.

### Statistical analyses

A descriptive analysis of the sample was carried out by describing the percentages for all variables and the mean and the standard deviation for the continuous variables. The database was analysed using SPSS Statistics software for Windows version 28.0 (IBM, Spain). Correlations between outcome and predictable variables were analysed using Pearson or Spearman tests, depending on the normality assessed by Shapiro-Wilk test. For statistically significant correlations, 95% confidence intervals were calculated. Statistical significance of the results was assumed as high (*p* < 0.001) or medium (0.001 < *p* < 0.05). Receiver Operating Characteristic (ROC) curve analyses was conducted to determine the optimal cut-off points for the TyG index and PA in discriminating adolescents according to glycemic control, cardiovascular risk, and body composition. Gold standard variables representative of each category will be used as reference standards. The area under the curve (AUC) was calculated to evaluate the predictive performance of each biomarker and interpreted as poor (0.5–0.6), moderate (0.6–0.7), acceptable (0.7–0.8), good (0.8–0.9) and excellent (≥ 0.9). Optimal cut-off values will be identified using Youden’s index to maximize sensitivity and specificity.

### Declaration of generative AI in scientific writing

During the preparation of this manuscript, the authors used Gemini to refine the language and improve readability. After using this tool, the authors reviewed and edited the content as needed and take full responsibility for the final manuscript.

## Results

### Sample characteristics

Table 1 summarizes the sociodemographic, anthropometric and body composition characteristics of the 73 participants (46.6% female, mean age 13.9 ± 2.5 years). Most participants had a normal weight based on BMI (67.1%) and both percentile and z-score criteria (56.3% each). Based on BMI, 19.2% were categorized as with overweight or obesity (15.1% and 4.1%, respectively), whereas using percentile or z-score criteria, 40.9% fell into these categories (23.9% overweight and 17% obesity). Mean PA was 6.2°, fat mass 18.1% (4.3 kg/m²), fat-free mass 81.1% (17.6 kg/m²), lean mass 77.6% (16.6 kg/m²) and skeletal muscle mass 43.6% (9.4 kg/m²). The mean adipose-muscular index was 0.5 kg/m², while the lean mass/fat mass ratio averaged 0.2. The ECW/TBW and ECW/ICW ratios had mean values of 0.4 and 0.6, respectively.

**Table 1:** Adolescents with type 1 diabetes mellitus’ socio-demographic, anthropometric and bioimpedance variables

	*n* (%)	Mean ± S
Gender	73 (100%)	
Male	39 (53.4%)	
Female	34 (46.6%)	
Age	73 (%)	13.9 ± 2.5
10 to 12 years old	20 (27.4%)	10.9 ± 0.8
13 to 15 years old	35 (47.9%)	13.9 ± 0.8
16 to 19 years old	18 (24.7%)	17.2 ± 1.4
BMI	73 (100%)	21.6 ± 3.9
< 18 Kg/m² (underweight)	10 (13.7%)	16.7 ± 0.9
18 to 24.9 Kg/m² (normal weight)	49 (67.1%)	20.7 ± 1.8
25 to 29.9 Kg/m² (overweight)	11 (15.1%)	27.1 ± 1.3
> 30 Kg/m² (obesity)	3 (4.1%)	32.1 ± 0.4
Percentile (BMI for age)	71 (97.2%)	66.8 ± 27.7
< P5 (underweight)	2 (2.8%)	2.8 ± 2.8
P5 to P84.9 (normal weight)	40 (56.3%)	54.3 ± 21
P85 to P94.9 (overweight)	17 (23.9%)	90.9 ± 2.7
≥P95 (obesity)	12 (17%)	98.3 ± 1.5
Z-score (BMI for age)	71 (97.2%)	0.71 ± 1.2
< −2,00 (underweight)	2 (2.8%)	−2.4 ± 0.1
−2,00 to + 0,99 (normal weight)	40 (56.3%)	0.1 ± 0.6
+ 1,00 to 1,99 (overweight)	17 (23.9%)	1.4 ± 0.3
≥ 2,00 (obesity)	12 (17%)	2.6 ± 0.4
PA (º)	65 (89%)	6.2 ± 1.6
Fat mass (Kg)	65 (89%)	11.4 ± 8.7
Fat mass (%)	65 (89%)	18.1 ± 10.8
Fat mass index (Kg/m^2^)	65 (89%)	4.3 ± 3.3
Fat free mass (Kg)	65 (89%)	47.8 ± 10.6
Fat free mass (%)	65 (89%)	81.8 ± 10.9
Fat free mass index (Kg/m^2^)	65 (89%)	17.6 ± 2.0
Lean mass (Kg)	65 (89%)	45.3 ± 10.1
Lean mass (%)	65 (89%)	77.6 ± 10.5
Lean mass index (Kg/m^2^)	65 (89%)	16.6 ± 1.9
Skeletal muscle mass (Kg)	65 (89%)	25.6 ± 6.5
Skeletal muscle mass (%)	65 (89%)	43.6 ± 6.2
Skeletal muscle mass index (Kg/m^2^)	65 (89%)	9.4 ± 1.4
Fat mass/lean mass ratio	65 (89%)	0.2 ± 0.2
Adipose-Muscular index (Kg/m^2^)	65 (89%)	0.5 ± 0.3
Total body water (L)	65 (89%)	34.3 ± 7.6
Intracellular water (L)	65 (89%)	20.9 ± 4.8
Extracellular water (L)	65 (89%)	13.4 ± 2.9
ECW/TBW	65 (89%)	0.4 ± 0.1
ECW/ICW	65 (89%)	0.6 ± 0.6

BMI: Body mass index; PA: Phase angle (50Hz); ECW/TBW: Extracellular water/Total body water; ECW/ICW: Extracellular water/Intracellular water

Table 2 presents glycemic control and biochemical variables. Most of participants used CGM (90.4%) and exhibited suboptimal glycemic control, with 75% having a CV > 36%, 68.2% a TIR < 70% and 80.6% an HbA1c > 6.5%. Mean biochemical values were as follows: glucose 162.9 mg/dL, total cholesterol 164.1 mg/dL, LDL 92.6 mg/dL, HDL 59.5 mg/dL, triglycerides 67.6 mg/dL, TyG index 8.4, LDL/HDL ratio 1.6, total cholesterol/HDL ratio 2.8, and triglycerides/HDL ratio 1.2.

**Table 2:** Adolescents with type 1 diabetes mellitus’ glycemic control and biochemical variables

	*n* (%)	Mean ± SD
Use of Continuous Glucose Monitor	73 (100%)	
Yes	66 (90.4%)	
No	7 (9.6%)	
CV	64 (87.6%)	40.9 ± 10.8
≤ 36% (controlled)	16 (25%)	30.4 ± 5.3
> 36% (not controlled)	48 (75%)	44.4 ± 9.9
TIR	66 (90.4%)	59.6 ± 16.9
≥ 70% (controlled)	21 (31.8%)	80 ± 6.7
< 70% (not controlled)	45 (68.2%)	50.1 ± 10.6
HbA1c	72 (98.6%)	7.4 ± 1.5
≤ 6.5% (controlled)	14 (19.4%)	5.9 ± 0.4
> 6.5% (not controlled)	58 (80.6%)	7.7 ± 1.4
Glucose (mg/d)	64 (87.7%)	162.9 ± 69
Total cholesterol (mg/dL)	63 (86.3%)	164.1 ± 36.8
LDL cholesterol (mg/dL)	55 (75.3%)	92.6 ± 28
HDL cholesterol (mg/dL)	59 (80.8%)	59.5 ± 14.6
Triglycerides (mg/dL)	65 (89%)	67.6 ± 34.5
TyG index	64 (87.7%)	8.4 ± 0.6
LDL/HDL	58 (80.6%)	1.6 ± 0.7
Total cholesterol/HDL	59 (80.8%)	2.8 ± 0.8
Triglycerides/HDL	59 (80.8%)	1.2 ± 0.8

CV: Coefficient of variation; TIR: Time in range; HbA1c: Glycosylated haemoglobin; TyG index: Triglyceride-glucose index

### Biomarker analysis

Table 3 shows the correlations of PA and TyG with predictable variables. Although PA and the TyG index were not significantly associated with each other (*p* = 0.958), both demonstrated several correlations with variables related to cardiovascular risk, body composition and glycemic control. PA was positively correlated with lean mass (Kg), lean mass index, skeletal muscle mass (Kg), fat-free mass (Kg) and fat-free mass index. Additionally, PA exhibited positive correlations with total cholesterol, LDL, HDL and age (*p* < 0.001). Conversely, the TyG index was negatively associated with lean mass (%), fat-free mass (%) and nearly exhibited a negative correlation with skeletal muscle mass (%) (*p* = 0.053). In addition, it showed a positive association with markers of adiposity such as fat mass (Kg and %), fat mass index, fat mass/lean mass ratio, adipose-muscular ratio, BMI, BMI percentile and BMI z-score. Furthermore, it correlated with triglyceride-related parameters, including blood triglyceride levels and the triglycerides/HDL ratio. Regarding glycemic control, PA did not show significant correlations with any glycemic variables. In contrast, the TyG index was positively associated with blood glucose levels, HbA1c, and CV, while showing a negative correlation with TIR. Additionally, age correlated positively with CV (*p* = 0.002).

**Table 3:** Phase angle and triglyceride-glucose index correlations in adolescents with type 1 diabetes mellitus

	PA			TyG index		
	Whole sample	*p* value	CI_95%_	Whole sample	*p* value	CI_95%_
Lean mass (Kg)	0.323 (*n* = 65)	0.009*	0.079–0.531	0.155 (*n* = 56)	0.255	
Lean mass (%)	0.141 (*n* = 65)	0.263		−0.335 (*n* = 56)	0.012*	−0.550- −0.079
Lean Mass index	0.334 (*n* = 65)	0.007*	0.090–0.539	0.221 (*n* = 56)	0.101	
Skeletal muscle mass (Kg)	0.250 (*n* = 65)	0.045*	−0.001-0.471	0.174 (*n* = 56)	0.199	
Skeletal muscle mass (%)	0.105 (*n* = 65)	0.404		−0.260 (*n* = 56)	0.053	
Skeletal muscle mass index	0.206 (*n* = 65)	0.100		0.251 (*n* = 56)	0.062	
Fat free mass (Kg)	0.308 (*n* = 65)	0.012*	0.062–0.519	0.158 (*n* = 56)	0.245	
Fat free mass (%)	0.107 (*n* = 65)	0.396		−0.337 (*n* = 56)	0.011*	−0.551- −0.082
Fat free mass index	0.293 (*n* = 65)	0.018*	0.045–0.506	0.227 (*n* = 56)	0.092	
Fat mass (Kg)	0.032 (*n* = 65)	0.800		0.368 (*n* = 56)	0.005*	0.109–0.581
Fat mass (%)	−0.089 (*n* = 65)	0.481		0.300 (*n* = 56)	0.025*	0.040–0.522
Fat mass index	−0.021 (*n* = 65)	0.868		0.354 (*n* = 56)	0.007*	0.093–0.570
Fat mass/lean mass ratio	−0.079 (*n* = 65)	0.532		0.325 (*n* = 56)	0.015*	0.060–0.547
Adipose-muscular ratio	−0.073 (*n* = 65)	0.564		0.307 (*n* = 56)	0.022*	0.040–0.533
BMI	0.057 (*n* = 65)	0.653		0.419 (*n* = 64)	0.001**	0.193–0.603
Percentile	−0.167 (*n* = 61)	0.198		0.471 (*n* = 60)	0.001**	0.239–0.652
Z-score	0.176 (*n* = 63)	0.168		0.420 (*n* = 62)	0.001**	0.183–0.611
Age	0.422 (*n* = 65)	0.001**	0.191–0.608	0.186 (*n* = 64)	0.140	
Glucose	0.052 (*n* = 56)	0.703		0.751 (*n* = 64)	0.001**	0.616–0.844
Total Cholesterol	0.310 (*n* = 55)	0.021*	0.041–0.538	0.204 (*n* = 63)	0.109	
HDL	0.348 (*n* = 51)	0.012*	0.072–0.575	−0.195 (*n* = 59)	0.139	
LDL	0.295 (*n* = 48)	0.042*	0.003–0.540	0.173 (*n* = 55)	0.208	
Triglycerides	−0.011 (*n* = 57)	0.937		0.705 (*n* = 64)	0.001**	0.555–0.810
Triglycerides/HDL	−0.175 (*n* = 51)	0.220		0.667 (*n* = 59)	0.001**	0.490–0.791
LDL/HDL	0.065 (*n* = 51)	0.653		0.120 (*n* = 57)	0.373	
Total Cholesterol/HDL	−0.074 (*n* = 51)	0.608		0.245 (*n* = 59)	0.061	
HbA1c	−0.182 (*n* = 64)	0.150		0.582 (*n* = 61)	0.001**	0.380–0.731
CV	0.073 (*n* = 59)	0.587		0.306 (*n* = 57)	0.023*	0.036–0.534
TIR	0.148 (*n* = 59)	0.265		−0.294 (*n* = 57)	0.027*	−0.515- −0.036

PA: Phase angle; BMI: Body mass index; HbA1c: Glycosylated haemoglobin; CV: Coefficient of variation; TIR: Time in range; *p < 0.05; **p < 0.01

### Cut-off points estimation

**Fig. 1:**
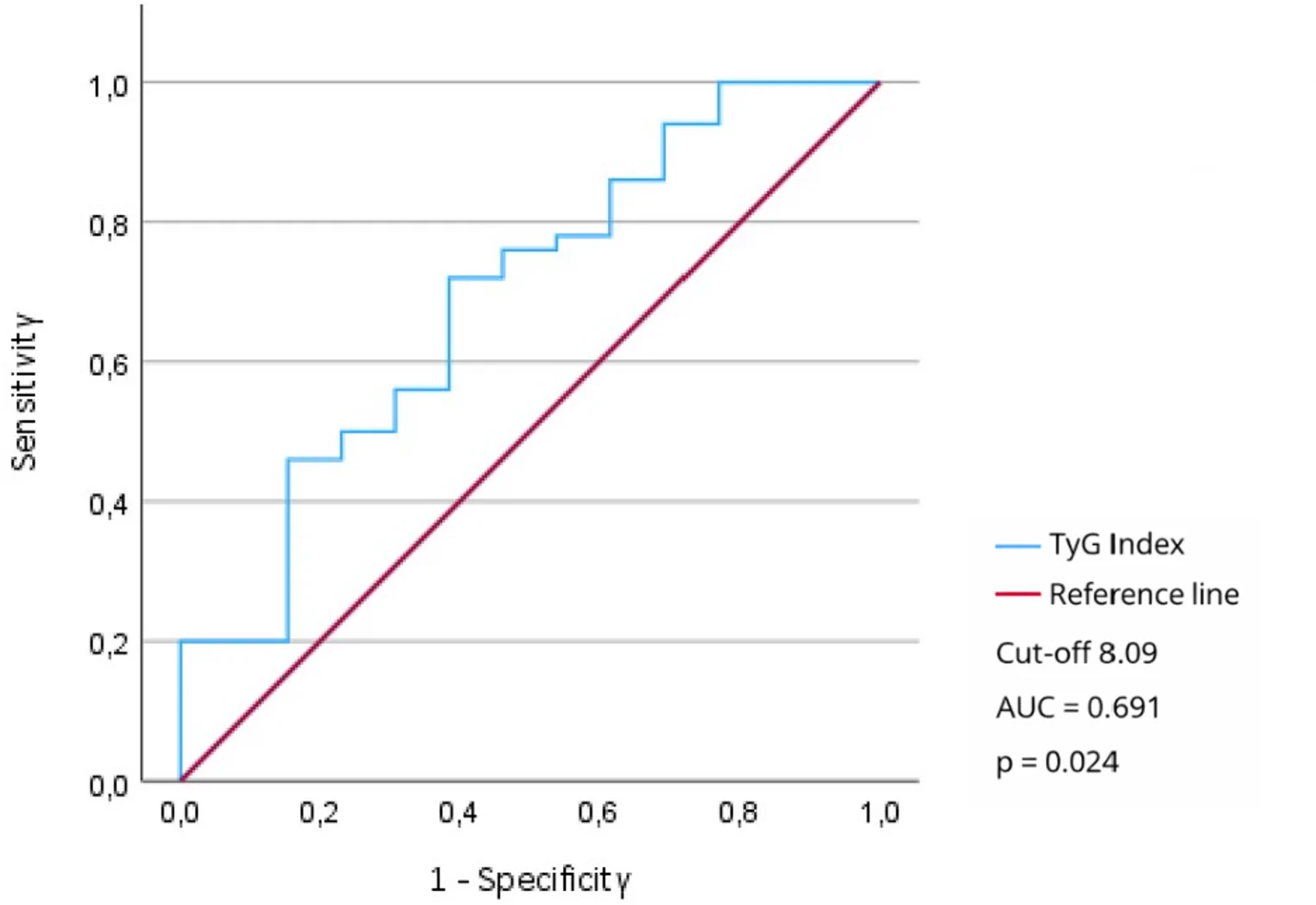
ROC curve of the TyG index for identifying suboptimal glycemic control (HbA1c > 6.5%) in adolescents with type 1 diabetes mellitus

This analysis aimed to establish cut-off points for the TyG index and PA to discriminate adolescents with T1DM based on glycemic control, cardiovascular risk and body composition. HbA1c was selected as the gold standard for glycemic control. Due to the lack of validated reference values for cardiovascular and body composition outcomes in this population, cut-off points could not be determined for either biomarker in these domains. Similarly, PA showed no significant associations with glycemic control, precluding the calculation of a meaningful threshold. Consequently, only a TyG cut-off related to HbA1c was derived. ROC curve analysis was performed, resulting in an AUC of 0.691 (CI_95%_:0.525–0.856; *p* = 0.024). This indicates that the TyG index has a moderate discriminatory capacity for identifying adolescents with suboptimal glycemic control. To determine the optimal cut-off point, Youden’s Index was applied. This analysis provided a cut-off value of 8.09 for the TyG index, which provided the best balance between sensitivity and specificity for identifying poor glycemic control in this population. The ROC curve illustrating the discriminatory performance of the TyG index is shown in Fig. 1.

## Discussion

This cross-sectional study aimed to further explore the potential utility of the TyG index and PA as biomarkers for metabolic risk assessment in adolescents with T1DM, specifically in relation to glycemic control, cardiovascular risk and body composition. Our findings revealed distinct associations between these biomarkers and parameters related to these key metabolic dimensions.

On one hand, the TyG was significantly associated with glycemic control, showing positive correlation with HbA1c and CV and a negative correlation with TIR, indicating poorer control at higher values and supporting its role as a biomarker of insulin resistance and predictor of metabolic dysfunction in this cohort [16–20].

Moreover, in our study the TyG index also correlated strongly with cardiovascular risk factors, including the fat mass/lean mass and triglycerides/HDL ratios, and with elevated triglycerides, consistent with prior evidence linking it to cardiovascular disease in adults [33] and to cardiovascular risk and insulin resistance in healthy children and adolescents [34]. Regarding body composition, the TyG index showed strong positive correlations with adiposity markers including fat mass, fat mass index, fat mass/lean mass ratio, BMI, BMI percentile and BMI z-score, indicating its role as an indicator of increased fat mass and cardiovascular risk, in line with findings in adults [35]. Identifying these components in clinical practice is particularly relevant during adolescence, an optimal period to establish the foundations of health behaviours that mitigate long-term metabolic risk [1], given the strong association between obesity and T1DM [4].

The interpretation of the TyG index in adolescents with T1DM requires particular caution. Although the TyG index has been extensively validated as a surrogate marker of peripheral insulin resistance in T2DM and non-diabetic populations [16, 17, 19, 20], its pathophysiological meaning in T1DM is more complex. In individuals with T1DM, hyperglycemia and dyslipidemia often coexist as a consequence of suboptimal insulin replacement, increased glycemic variability and the metabolic effects of puberty [36]. As a result, TyG values in this population may partly reflect the degree of metabolic control rather than true peripheral insulin resistance. This distinction is relevant, as insulin resistance in T1DM arises from mechanisms that differ from those in T2DM, such as exogenous insulin dosing, hormonal changes during puberty and glycemic instability, making the TyG index a potentially indirect marker [36, 37]. Therefore, its use in T1DM should be contextualized within the broader metabolic profile [36] and future studies incorporating direct measures of insulin sensitivity are needed to clarify its physiological interpretation in this population.

On the other hand, in our study PA did not exhibit significant correlations with glycemic control, contrasting with findings in T2DM [38–40]. This may be related to the specific population studied as well as the sample number, as the availability of adolescent participants with T1DM in hospital settings is more limited. However, our sample size remains consistent with those of other studies conducted in this population [24, 41]. Regarding cardiovascular risk, PA correlated positively with lean mass index, a biomarker of cardiovascular protection, consistent with evidence of its inverse association with cardiovascular disease in adults [23, 42]. Interestingly, PA was also positively associated with total cholesterol, LDL and HDL, which may reflect the complex relationship between muscle mass and lipid metabolism, whereby greater muscle mass enhances lipid turnover and transport, leading to transient cholesterol elevations [43].

PA correlated positively with skeletal muscle mass, skeletal muscle mass index, fat-free mass and fat-free mass index, indicating its potential role as a biomarker of favorable body composition since muscle and lean mass integrity are crucial for metabolic health [21]. These findings align with previous evidence linking PA to nutritional status and prognosis across various clinical populations [22]. As a rapid, simple, reproducible, precise and reliable measure, PA can be applied across in diverse clinical settings, including healthy individuals and those with diverse pathologies [21], and allows monitoring of short-term changes in body composition. Current evidence further suggests that PA may outperform other nutritional, biochemical, and anthropometric markers, such as BMI, in predicting morbidity and mortality [22].

We consider important to point out the limitations of relying solely on BMI to assess body composition in adolescents with T1DM, as is commonly performed in routine clinical practice [44]. BMI does not differentiate between fat mass and lean mass, potentially misclassifying obesity and underestimating metabolic risk [44]. Accurate obesity diagnosis is crucial given its impact on insulin resistance and cardiovascular risk [13, 44]. The higher proportion of participants classified as overweight/obese using the percentile and z-score criteria compared to BMI, reinforces the need for more precise assessments beyond traditional anthropometry measures. In this context, BIA analysis and its derived parameters, including PA, provide a more accurate evaluation of body composition [13, 45] by distinguishing between lipid mass (commonly referred to as fat mass) and lipid-free mass (often referred to as fat-free mass) within the molecular model [46]. This compartmental approach provides a biologically grounded understanding of metabolic profiles, supporting tailored clinical decisions and improving metabolic risk assessment in adolescents with T1DM [13, 45, 46].

We employed body composition indices to account for age-related variability and physical development among participants. However, their interpretation remains challenging due to the absence of validated cut-off points in this population, as current thresholds are derived from adults and may not be directly applicable [29, 47]. Caution is therefore warranted, and further research is needed to establish adolescent-specific criteria. Given the adolescent cohort, age emerged as a significant factor influencing metabolic parameters: PA increased with age, reflecting normal development [45]. However, age was also associated with poorer glycemic control, as evidenced by higher CV and lower TIR. Thus, managing T1DM may become challenging during puberty, potentially due to hormonal changes and lifestyle factors [3, 48, 49].

In parallel, the rapid evolution of CGM technology should also be considered when interpreting glycemic metrics in adolescents with T1DM. New intermittently scanned CGM systems have shown performance comparable to established sensors and may influence the assessment of TIR, variability and other CGM-derived parameters [50]. As new devices continue to emerge, differences in analytical performance and usability may influence CGM-derived metrics, which reinforces the importance of interpreting these parameters within the technological context in which they are obtained.

The establishment of specific cut-off points for PA and TyG index in adolescents with T1DM is essential for accurately assessing metabolic risk. Currently, no validated reference values exist for these biomarkers in this population, limiting their clinical applicability. Tentative cut-off points have been proposed for healthy adolescents, but their extrapolation to T1DM is questionable given metabolic differences. For the TyG index, few studies in non-diabetic adolescents have proposed thresholds, with no consensus: one study [51] reported 4.21 in prepubertal children and 4.33 in pubertal adolescents, while another [15] in Brazilian adolescents proposed higher thresholds, 7.91 for boys and 7.94 for girls. In both cases, most of our sample would exceed these values, likely due to diabetes-related dysregulation. Regarding PA, although it is a recognized marker of cellular integrity and nutritional status, no reference values exist for adolescents with T1DM. In healthy individuals, PA ranges between 5.5° and 9° [52], but it varies by age, sex and health status [45, 52], making direct application to our cohort inappropriate. Further research is needed to stablish T1DM-specific cut-offs that optimize predictive value and clinical utility.

Our study proposes a preliminary TyG index cut-off for glycemic control in adolescents with T1DM. Although the ROC analysis provided a moderate AUC of 0.691, it should be considered that this value is close to the acceptable predictive performance. However, this cut-off should be interpreted with caution. To our knowledge, this is the first attempt to establish a TyG index threshold in this population, and therefore even a modest AUC represents a meaningful starting point. The present threshold offers an initial framework that future studies can refine and validate. It should also be noted that our sample was relatively homogeneous and predominantly composed of participants with poor glycemic control, which may limit generalizability. Future research should include larger, more heterogeneous cohorts, ideally stratified by age and sex, to refine and validate cut-offs for both TyG index and PA, enhancing their clinical utility.

Both biomarkers are valuable for assessing obesity, glycemic control and cardiovascular risk, complementing each other as they capture different aspects. Ideally, clinical evaluations would incorporate both biomarkers for a more comprehensive assessment. Nevertheless, the TyG index stands out as particularly practical for routine use, relying on commonly available measurements without additional equipment [38].

Despite providing valuable insights, this study has several limitations. Its cross-sectional design prevents causal inference between TyG index, PA and metabolic risk, highlighting the need for longitudinal studies. Although BIA offers more detailed body composition assessment than BMI, its accuracy can be affected by hydration status, recent fluid intake or bladder voiding [52, 53]. Controlling these variables was challenging due to the limited time available and the variability in appointment schedules, though participants received standardized instructions prior to measurement.

Additionally, another important limitation is the absence of pubertal staging. As puberty is a major physiological determinant of insulin resistance, body composition and lipid profile, its omission may have introduced residual confounding in the observed associations. Although pubertal development was considered conceptually, its substantial interindividual variability in adolescents with T1DM made standardized classification unfeasible in our setting and would have resulted in comparison groups too small to yield statistically meaningful conclusions. Future studies should incorporate validated pubertal assessment to better contextualize metabolic and body composition markers in this population. It should also be noted that physical activity levels and dietary quality were not assessed in this study. Both factors strongly influence muscle mass, lipid metabolism and phase angle, and could therefore partially explain the positive associations observed between PA, total cholesterol and LDL. The absence of these variables limits our ability to determine whether these correlations reflect physiological adaptations related to body composition or are confounded by lifestyle behaviors.

It is also important to acknowledge the absence of data on smoking habits, alcohol consumption and recreational drug use. Although the mean age of the cohort was relatively young, these behaviours may be present in older adolescents and could influence metabolic parameters, including lipid profile, insulin resistance and body composition. These variables were not systematically recorded in clinical charts and therefore could not be incorporated into the analyses. Another relevant aspect is that total daily insulin dose and diabetes duration were not considered in the analyses. Both variables are clinically relevant and may influence TyG values, lipid profile, glycemic control and body composition. Incorporating these parameters in future analyses would help better characterize their potential impact on metabolic and body composition markers in adolescents with.

In addition, data on β-cell function derived from oral minimal model analyses (such as φ total) were not available in our cohort. Recent evidence suggests that φ total may predict stage progression in T1DM [54] and exploring its relationship with TyG or PA could have provided further insight into the interplay between insulin secretion, metabolic control and body composition in adolescents. However, these measurements are not routinely performed in clinical practice and therefore could not be incorporated into our analyses. Finally, no assessment of collinearity among predictive variables was conducted, which may influence the stability of multivariable models. Future studies should address these limitations to strengthen predictive analyses.

## Conclusion

Our study highlights the complementary roles of the TyG index and PA in assessing metabolic risk in adolescents with T1DM. The TyG index effectively reflects adiposity, glycemic variability and cardiovascular risk, while PA is a robust indicator of body composition, specifically of muscle mass. The TyG index, based on routine laboratory parameters, is feasible as a primary care screening tool, whereas PA requires BIA devices, limiting its accessibility. Integrating both biomarkers into clinical practice could enhance risk stratification and enable timely interventions to optimize glycemic control, body composition and mitigate long-term cardiovascular complications. Future longitudinal and intervention-based studies are needed to validate these associations and explore targeted strategies for improving metabolic health in this population.

## Data Availability

The data supporting this study are available in Zenodo at the following link: https://zenodo.org/records/19074127. Additional data are available from the corresponding author upon reasonable request.
